# A22 USING INTESTINAL ORGANOIDS TO CHARACTERIZE THE NAIP-NLRC4 INFLAMMASOME RESPONSE IN THE INTESTINAL EPITHELIUM

**DOI:** 10.1093/jcag/gwac036.022

**Published:** 2023-03-07

**Authors:** A Ranger, S Girardin

**Affiliations:** Laboratory Medicine and Pathobiology, University of Toronto, Toronto, Canada

## Abstract

**Background:**

The NAIP-NLRC4 inflammasome is an important innate defence mechanism in intestinal epithelial cells (IECs) that protects the gut from invasive pathogens. NAIP-NLRC4 activation triggers pyroptotic cell death, releases active interleukin (IL)-18, and promotes the expulsion of infected enterocytes. Dysregulated inflammasomes result in exaggerated inflammation of the intestinal mucosa which is characterisitic of inflammatory bowel disease (IBD). There is increasing evidence that inflammasomes in IEC behave differently than in immune cells. However, a majority of inflammasome pathway characterization is studied immune cells. In addition, NAIP-NLRC4 activity in macrophages has been shown to be important for regulating the expression of nitric oxide (*Nos2*). The potential transcriptomic impact of NAIP-NLRC4 activity in IECs has yet to be explored.

**Purpose:**

We will use intestinal orgnaoids to systematically characterize the NAIP-NLRC4 inflammasome pathway and identify the transcriptomic impact of NAIP-NLRC4 activity in IECs. To better define the role of NAIP-NLRC4 activity during a physiological infection, we have developed a novel *ex vivo* model of *Shigella* infection in 3D organoids.

**Method:**

Organoids were derived from the ileal crypts of wild type (WT) and *Nlrc4*^*-/*-^, *Casp1*^*-/*-^, *Pycard*^*-/*-^, or *Tlr5*^*-/*-^ mice and stimulated with Pam3CSk4, LPS, or flagellin. Inflammasome activation was assessed by Western blot (WB) and propidium iodide uptake. WT and *Nlrc4*^*-/*-^ organoids were infected with WT or a non-invasive *Shigella* mutant and the inflammasome response was evaluated by WB and a colony forming unit assay. WT and *Nlrc4*^*-/*-^ organoids were stimulated with flagellin and gene expression was assessed by RT-qPCR.

**Result(s):**

Basolateral organoid stimulation with bacterial ligands revealed a novel response of IECs to bacterial flagellin that results in pyroptosis and IL-18 processing. Basolateral internalization of flagellin occurred in a TLR5-independent manner. Inflammasome activation by flagellin was fully abrogated in *Nlrc4*^*-/*-^ and *Casp1*^*-/*-^ organoids while only IL-18 processing was affected in *Pycard*^*-/*-^ organoids. Infection with only WT *Shigella* induced inflammasome activation in an NLRC4-dependent manner. Interestingly, flagellin stimulation of WT but not *Nlrc4*^*-/*-^ organoids led to increased expression of *Nos2.* Furthermore, *Nlrc4*^*-/*-^ organoids had significantly lower expression levels of cytokine genes *Ccl20*, *Cxcl1*, and *Tnf* following inflammasome activation.

**Conclusion(s):**

Our study demonstrates an integral role for epithelial NAIP-NLRC4 inflammasomes in the response to bacterial flagellin and *Shigella* infection. We have uncovered a novel response of IECs to basolateral flagellin stimulation and revealed that NAIP-NLRC4 is important for the transcriptional regulation of inflammatory genes. Further work will examine how NAIP-NLRC4 activation controls inflammation and epithelial integrity by analyzing the NLRC4-dependent transcriptome and the effects on cell proliferation, differentiation, and barrier integrity.

**Please acknowledge all funding agencies by checking the applicable boxes below:**

CCC, CIHR

**Disclosure of Interest:**

None Declared

